# Revolutionizing Playing with Skeleton Atoms: Molecular Editing Surgery in Medicinal Chemistry

**DOI:** 10.2174/0113895575316229240611113946

**Published:** 2024-06-12

**Authors:** Amgad M. Rabie

**Affiliations:** 1 Head of Drug Discovery & Clinical Research Department, Dikernis General Hospital (DGH), Magliss El-Madina Street, Dikernis City 35744, Dikernis, Dakahlia Governorate, Egypt;; 2 Pharmaceutical Chemistry Department, Faculty of Pharmacy, Ain Shams University, Abbassia, 11566, Cairo, Egypt

**Keywords:** Skeletal editing (SKED) strategy, synthetic organic chemistry, molecular drug design, drug discovery, medicinal chemistry, artificial intelligence (AI), chemico-editing machine, chemical robot, heterocyclic compound, carbon/nitrogen/oxygen atom

## Abstract

Finding the most perfect drug candidates in the fields of drug discovery and medicinal chemistry will remain the main interest of drug designers. This concern necessitates organic and medicinal chemists, in most examples, to precisely design and search for drug candidates that are very analogous to the present effective drugs with solving, mainly, their proven critical pharmacological and clinical issues through slightly changing one or two atoms of the principal functional skeletons of the molecules of these present therapeutics by atom swapping, removal, and/or addition procedures in organic chemical synthesis. This accurate modern chemicosimilarity tactic in drug discovery surely saves time while keeping us very close, or sometimes highly superior, to the parent pharmacophoric bioactivity (*i.e.*, keeping considerable analogy to the parent therapeutic molecule). From this perspective and logic, the science of skeletal editing of molecules (*i.e.*, skeletal molecular editing) arose in the era of artificial intelligence (AI) and its dramatic predictions. As a pioneer in this modern branch in pharmaceutical and therapeutic organic chemistry, in this up-to-date minireview and perspective article, an attempt was made to introduce skeletal editing and its synthetic surgeries (over molecules) to the audience (including irrelevant readers) in a simpler and more attractive way as a novel chemical technology, highlighting the previous synthetic trials (in general), demonstrating the three main techniques, and, finally, discussing the future therapeutic needs and scenarios from a medicinal chemist's viewpoint.

## 1 INTRODUCTION

Organic chemistry appears to the beginners and uninformed to be a bewildering display of Egyptian hieroglyphs and a whirlwind of zigzags and hexagons spinning over the page. Since my first undergraduate years in the Faculty of Pharmacy (Mansoura University, Egypt), I have been dreaming of establishing a new pharmaceutical chemistry branch comprising unique techniques in organic chemistry that will be concerned with all dreamed aspects and visions at the atomic level of the substances. One of my dreams was to reach each atom in the molecule at its position, interact with it, and decide its planned destiny, *i.e.*, to change the organic reaction game from the external molecular level to the internal atomic level. Later on, as a medicinal chemist, the ultimate goal of this dream was to increase the ability of relevant chemists to accurately edit chemical molecules by replacing, adding, or deleting single atoms in their cores/scaffolds through extremely delicate chemical surgeries or reactions.

**Fig. (1):**
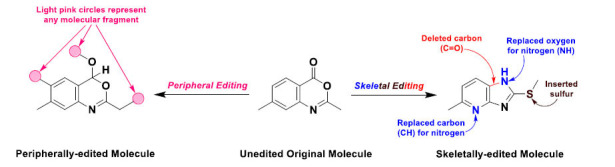
A scheme demonstrating modern skeletal molecular editing (on the right side) *versus* classical peripheral molecular editing (on the left side) on a representative molecule in organic and medicinal chemistries.

In general, normal classical organic reactions occur at the peripheries (*e.g.*, substituents) of interacting molecules to form new larger molecules without affecting the internal backbones or cores (traditional peripheral molecular editing) (Fig. **1**). While on the other hand, our revolutionary dream is to play with and modify the major cores of molecules through designed chemical nanosurgeries or, sometimes, picosurgeries, using even robo-chemists and drug-maker machines for these types of precise and difficult organic synthesis (revolutionary skeletal molecular editing or, simply, skeletal editing; I suggest officially abbreviating it in the coming days as “SKED”, *e.g.*, SKED strategy) (Fig. **1**). These molecular nanosurgeries partially depend on the “moonshot” pathway concept. As an explosion in organic reaction methodologies, this type of magical molecular nanosurgery may completely alter how organic chemists create compounds and may greatly speed up the drug development/discovery process.

In the last few years, there have been some successful attempts to synthesize new molecules using this new technique unintendedly; however, most of these reactions were not effective with all chemical skeleton (core) types, could not bypass the interferences caused by many peripheral fragments, and were also almost not workable with less simple molecules [1]. Although skeletal or core editing was on the rise in both the years 2021 and 2022, it actually became an emerging independent branch capable of being standalone in pharmaceutical organic chemistry in the previous year, 2023 [1]. This was achieved by having the least sufficient deal of data, information, characteristics, and features that can systemically build up this new organic/medicinal chemistry branch that effectively complements the other branches of organic/medicinal chemistry.

## 2 PREVIOUS ORGANIC SYNTHETIC ATTEMPTS

**Fig. (2):**
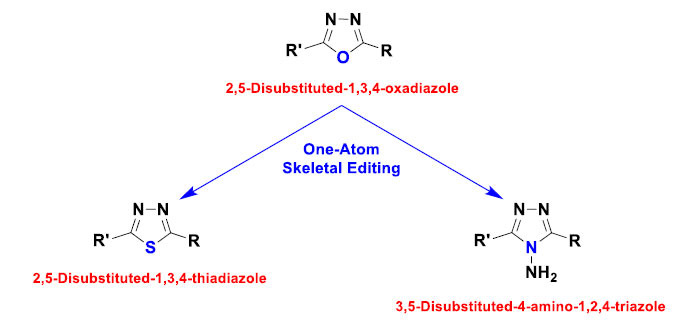
Skeletal editing of the scaffold of 1,3,4-oxadiazoles into the scaffolds of their analogs, 1,3,4-thiadiazoles and 1,2,4-triazoles.

**Fig. (3):**
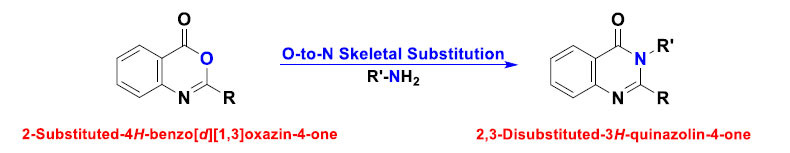
Skeletal editing of the scaffold of 4*H*-benzo[*d*][1,3]oxazin-4-ones into the scaffold of their analogs, 3*H*-quinazolin-4-ones.

In the last decade (2015-2024), I and my research teams carried out many successful syntheses of primary skeletal molecular editing (together with those of conventional peripheral molecular editing) [2-9]. Though most of these reactions showed direct actions on the principal scaffold moieties of the reactants (mainly, one- or two-atom modifications), they are still considered initial attempts of combined classic and advanced organic synthesis. A famous example of skeletal molecular editing is the substitution or replacement of the core oxygen atom of the 1,3,4-oxadiazole ring in 2,5-disubstituted-1,3,4-oxadiazoles, either with a core sulfur atom of the 1,3,4-thiadiazole ring in 2,5-disubstituted-1,3,4-thiadiazoles or with a core nitrogen atom of the 1,2,4-triazole ring in 3,5-disubstituted-4-amino-1,2,4-triazoles, depending on the reaction conditions (Fig. **2**) [2, 3]. Another known example (which was observed in several optimized drug design trials) is the replacement of the core oxygen atom of the 4*H*-benzo[*d*][1,3]oxazine ring in 2-substituted-4*H*-benzo[*d*][1,3]oxazin-4-ones with another core nitrogen atom of the 3,4-dihydroquinazoline ring in 2,3-disubstituted-3*H*-quinazolin-4-ones (Fig. **3**) [10]. In both examples, skeletal editing could also be called skeletal substitution.

## 3 TYPES OF SKELETAL OR CORE EDITING IN STRUCTURAL CHEMISTRY

The motif of atom replacement, deletion, or insertion in any chemical skeleton is a very dreamy magic idea in organic reactions and structural chemistry. Numerous methods for intramolecularly transferring individual atoms into and out of the skeletons of molecules have been developed by organic chemists. However, the current condensed examples (displayed in general style) may only apply to particular types of organic structures or require specific reaction circumstances, *i.e.*, skeletal editing in organic chemistry is not yet effective for all compounds and is still under progressive development, as previously stated. These chemical edits are carried out for molecules in organic chemistry for the sake of mainly easing or enabling certain types of organic chemical reactions (from a chemical point of view) and in medicinal chemistry for addressing mainly pharmacokinetic, pharmacological, metabolic, and toxicological issues (from a clinical point of view). Three major categories of skeletal editing reactions are currently present in organic chemistry laboratories, as follows:

### 3.1 Atom Replacement (Skeleton Analogism/Isosterism)

**Fig. (4):**
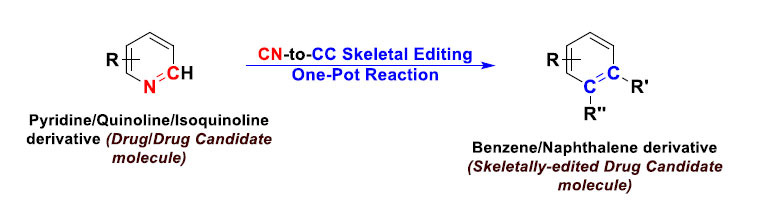
Direct skeletal editing of pyridine-core-containing molecules through atom-pair swap from CN to CC to produce benzene/naphthalene-core-containing molecules, respectively, in a modular manner (the reaction is a one-pot sequential reaction).

This type of skeletal editing comprises various kinds of atom replacement “substitution”/rearrangement reactions. Mainly, one carbon atom in the organic molecule scaffold is replaced by either an isotopic carbon atom, a nitrogen atom, an oxygen atom, or a sulfur atom (the reverse is also possible for the four atoms) [11, 12]. Sometimes, two atoms are replaced by two other atoms, *e.g.*, from NN to CC [12]. Another major example is the swapping of an oxygen atom for either one nitrogen atom or one sulfur atom [2, 3]. This approach of reactions was developed and applied to several kinds of late-stage modifications of drugs/drug candidates and their derivatives (*e.g.*, tropicamide, loratadine, stanolone, indomethacin, and probenecid), as in the case of the pyridine-editing strategy (Fig. **4**) [12].

### 3.2 Atom Deletion (Skeleton Narrowing/Straitening)

**Fig. (5):**
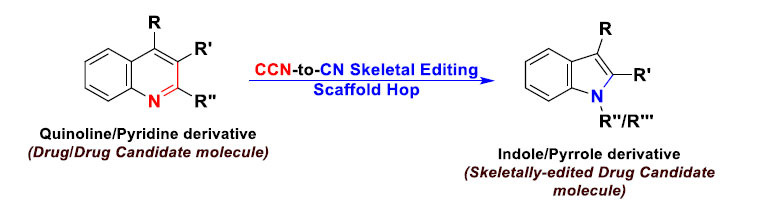
Comparative skeletal editing of quinoline/pyridine-core-containing molecules through scaffold hopping from CCN to CN to produce indole/pyrrole-core-containing molecules, respectively, mainly for drug-likeness comparisons in SAR studies (this approach could also be applied to pyrimidine-to-pyrazole and bipyridine-to-phenylpyrazole interconversions in drug design and optimization strategies).

This type of skeletal editing was primarily developed to enable drug discovery chemists to delete “remove” at least one carbon atom (or other specified atoms) from the drug/drug candidate molecule's skeleton to effectively interconvert this molecule to another more successful one during the structure-activity relationship (SAR) studies, *e.g.*, to decrease the molecular weight, contract the skeleton chain length, and/or adjust certain pharmacokinetic properties. An example of this skeleton contraction and scaffold hopping strategy is the interconversion between the two chemical classes, quinolines and indoles (Fig. **5**) [11-13]. Both of them are very common core motifs in drug molecules, and they differ only by a single carbon atom in their ring structures, making this ring-editing strategy between them very useful during the SAR studies, *e.g.*, when comparing the two members of the statin class of pharmaceuticals, the cholesterol-lowering therapeutics pitavastatin (the quinoline derivative) and fluvastatin (the indole analog) [13]. This strategy is also applied when comparing pyridine drugs and their skeleton-contracted pyrrole/pyrazole analogs, *e.g.*, the two antiinflammatory drugs etoricoxib and celecoxib; the same approach is also obvious when comparing compounds in a certain drug development series, *e.g.*, 5,10-dideazafolic acid (an inactive molecule) and pemetrexed (an active chemotherapeutic molecule) [12, 13].

### 3.3 Atom Insertion (Skeleton Widening/Expanding)

**Fig. (6):**
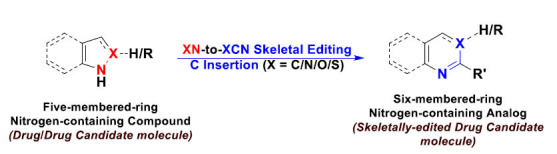
Synthetic skeletal editing of five-membered-core-containing molecules through carbon insertion by XN-to-XCN interconversions (where X atom is either carbon, nitrogen, oxygen, sulfur, or another atom) to produce six-membered-core-containing molecules; this molecular editing approach is usually used for the total synthesis of naturally occurring compounds and their synthetic/semisynthetic analogs (this approach could also be applied to bicyclic, tricyclic, and multicyclic molecules “containing nitrogenous heterocyclic five-membered rings” in drug discovery and medicinal chemistry strategies).

This type of skeletal editing is almost the reverse of the previous atom-removal strategy, and it also has the same importance in drug design and discovery tactics. This strategy enables medicinal chemists to insert “add” carbon, nitrogen, and/or oxygen atom(s) (or other specified atoms) to the drug/drug candidate molecule's skeleton to interconvert this molecule to another more effective one. This strategy is specifically very useful in the chemical retrosynthesis of bioactive natural products and their analogs from simple and available small-molecule precursors [14, 15]. Known examples of this skeleton expansion strategy are the pyrrole-to-pyridine, pyrazole-to-pyrimidine, indole-to-quinoline, indole-to-quinazoline, and indazole-to-quinazoline molecular editing conversions, *e.g.*, the total organic synthesis of the natural pyridine-containing alkaloids complanadine A and lycodine, as well as simplified derived analogs, from a simple starting material containing a five-membered pyrrole ring in its chemical structure (pyrrole-to-pyridine molecular editing strategy) (Fig. **6**) [11, 12, 14, 15].

## 4 MEETING THERAPEUTIC NEEDS

One of the major objectives of organic chemistry is to pour compounds of bioactive potential into the pool of therapeutic or medicinal chemistry. One could be tempted to compare skeletal editing to the highly popular gene-editing method CRISPR in biotechnology. It actually has poor similarity and comparison. CRISPR simply needs to handle the four nucleotides of DNA or RNA [16, 17]. On the other hand, skeletal editing is much more generalizable since the editing techniques here are far more broadly applicable and work on millions of diverse organic molecules (including organometallic compounds).

Interestingly, organic chemical skeletal editing could be considered a modern pathway for modifying pharmaceutically relevant bioactive molecules through mainly replacing a carbon atom for one atom of nitrogen, oxygen, sulfur, selenium, or others (and the other way around), *i.e.*, mainly interconversions of arenes and heteroarenes [18]. The produced analogs and derivatives might open up very important tracks of research in medicinal chemistry drives. These highly analogous derivatives might provide substantial solutions to pharmacodynamics/pharmacokinetics-related issues of the original medicines, *e.g.*, unfavorable pharmacokinetic profiles (including the metabolic pathways), low drug-likeness scores, severe side effects, and/or considerable toxic behaviors *e.g.*, [1, 18-20].

The development of much simpler and cheaper one-pot procedures of organic chemistry substitution (interconversion) methods between mainly the previously-mentioned atoms (carbon, nitrogen, oxygen, sulfur, and selenium atoms, in addition to phosphorus, silicon, boron, and arsenic atoms), *i.e.*, intended or targeted chemical mutations, would continue to push the limits of pharmaceutical organic chemistry, revealing chemical mechanisms that might serve as the foundation for upcoming substitution procedures of skeletal or core editing as a general attractive synthetic tactic to exchange influential atoms in medicinal chemistry. Continuous diverse contributions from the entire synthetic organic chemistry community worldwide could make it very possible to interchange influential atoms at whim inside the fundamental cores or scaffolds of pharmaceutically significant compounds.

## CONCLUDING FOUNDER OPINION AND FUTURE SCENARIOS

The dream of having extreme-technology chemico-editing machines (supposed to be composed of two principal parts: a software part, which will have a very complicated programmed electronic dry-lab system, connected to a hardware part, which will have a high-quality chemical reactor wet-lab system) with the controllable capacity to directly and precisely edit and modify organic chemical molecules at exactly their central cores to generate closely-related new molecules possessing, for instance, improved biological activities and reduced adverse effects is gradually approaching its reality. In my opinion, artificial intelligence (AI) methodologies will certainly play a considerable role in this mounting new technology of molecular editing. Synthetically, the skeletal editing strategies are based on the concept and fact that compounds of unsaturated open-chain, cyclic, or heterocyclic scaffolds (as well as their saturated analogs) could be interconverted to each other or even to themselves (the same type) but with different numbers of skeleton atoms, providing time-saving and site-directed drug discovery approaches.

Last but not least, in the era of molecular femtosurgeries, the applicability and practicality of the hundreds of methods of the various strategies for modern skeletal editing of heterocyclic (mainly heteroarene), cyclic (mainly arene), and open-chain (mainly alkane and alkene) cores will represent a very special importance in the next years in the fields of drug discovery and medicinal chemistry, for example, as in approaches for late-stage modifications and site-selective functionalizations in drug development, approaches for specific fragment coupling of complex molecules, approaches for detecting and revealing a drug's mechanism of action (*e.g.*, atom knockout approaches for deciphering the mechanistic chemicobiological interactions of drugs in the human body), approaches for SAR expansions and improvements, approaches for targeted drug repurposing, and approaches for adjustments of drug-likeness behaviors and profiles of therapeutics for gaining clinical benefits and solving clinical issues, concerns, and dangers. Undoubtedly, this new effective gate for drug design and development will provide drug discoverers and medicinal chemists with many successful tactics for editing organic molecules in lab flasks exactly as in note papers.

## SUMMARY

This exclusive minireview/perspective paper discusses the scope and principles of the emerging new science of skeletal editing (SKED) in molecular design, optimization, modeling, and modification as a new practicable branch in medicinal chemistry. The paper specifically sheds light on the revolutionary methods designed to improve the drug-likeness profiles of the targeted drug molecules by operating certain chemical femtosurgeries over skeletal atoms. Further, the paper also highlights the relevant general synthetic strategies, demonstrates the three main core-editing techniques, and, finally, discusses the future therapeutic needs and scenarios from the viewpoint of one of the founders of this organic chemistry branch.

## LIST OF ABBREVIATIONS

AI: Artificial Intelligence
SAR: Structure-Activity Relationship
SKED: Skeletal Editing
